# Supplementary material for: Causal Relationship between Aging and Anorexia Nervosa: A White-Matter-Microstructure-Mediated Mendelian Randomization Analysis
Source: Biomedicines. 2024 Aug 16;12(8):1874. doi: 10.3390/biomedicines12081874 (PMC11351342; doi:10.3390/biomedicines12081874)
Supplement: Supplementary file 1 [file biomedicines-12-01874-s001.zip › Supplementary Figure/Figure S8/Leaveoneout plot of MR analysis for TL to ubm-b-1477.pdf]

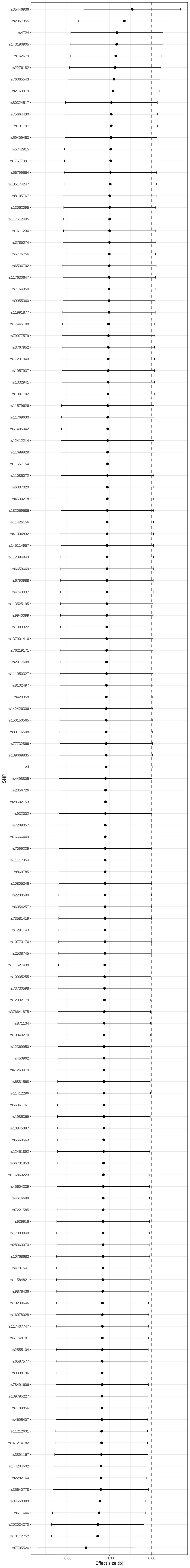
rs35446936
rs2967355
rs4724
rs143190905
rs762679
rs2276182
rs76065543
rs2763979
rs80324517
rs75664430
rs131797
rs59409453
rs5742915
rs17677991
rs56799554
rs185174247
rs8105767
rs13062095
rs117512405
rs1611236
rs3785074
rs6776756
rs6536702
rs117630647
rs7164950
rs9955360
rs11991877
rs17445108
rs79977579
rs3767952
rs77231040
rs1957937
rs1332941
rs1907702
rs11579626
rs11769630
rs61405042
rs12412214
rs11699829
rs11557154
rs11085072
rs6007020
rs4530278
rs182059586
rs11426156
rs41304832
rs145114957
rs112394943
rs6659669
rs6790988
rs4743037
rs113525195
rs9940099
rs1003322
rs137901416
rs76219171
rs2977608
rs111950327
rs8102497
rs429358
rs142426306
rs150150565
rs80116508
rs77732866
rs139669835
All
rs4498805
rs2056726
rs28502153
rs932002
rs7209057
rs76666449
rs7099229
rs11117354
rs869785
rs10805346
rs2230590
rs6054257
rs73581419
rs1291143
rs10773176
rs2538745
rs111527438
rs10905255
rs73730598
rs12932179
rs376641875
rs871134
rs10840270
rs12369950
rs450962
rs41269079
rs6881568
rs11412296
rs56061761
rs1985369
rs10845387
rs6669563
rs12451892
rs66731853
rs116863223
rs45604339
rs4616688
rs7221585
rs939916
rs17803849
rs28363070
rs10768683
rs4731541
rs11584821
rs9878436
rs13230646
rs16978028
rs117407747
rs61748181
rs2555104
rs6587577
rs9398196
rs78491606
rs139795227
rs7790856
rs4695407
rs11212631
rs141214782
rs3891167
rs144204502
rs2282764
rs35640778
rs34550383
rs611646
rs202034370
rs10112752
rs7705526
SNP
−0.06
−0.03
0.00
Effect size (b)
